# The Complex Story of Plant Cyclic Nucleotide-Gated Channels

**DOI:** 10.3390/ijms22020874

**Published:** 2021-01-16

**Authors:** Edwin Jarratt-Barnham, Limin Wang, Youzheng Ning, Julia M. Davies

**Affiliations:** Department of Plant Sciences, University of Cambridge, Cambridge CB2 3EA, UK; ecj39@cam.ac.uk (E.J.-B.); lw577@cam.ac.uk (L.W.); yn283@cam.ac.uk (Y.N.)

**Keywords:** calcium signalling, CaM, calmodulin, cAMP, cGMP, CNGC, cyclic nucleotide-gated channel, CNGCL, cyclic nucleotide-gated channel like

## Abstract

Plant cyclic nucleotide-gated channels (CNGCs) are tetrameric cation channels which may be activated by the cyclic nucleotides (cNMPs) adenosine 3′,5′-cyclic monophosphate (cAMP) and guanosine 3′,5′-cyclic monophosphate (cGMP). The genome of *Arabidopsis thaliana* encodes 20 CNGC subunits associated with aspects of development, stress response and immunity. Recently, it has been demonstrated that CNGC subunits form heterotetrameric complexes which behave differently from the homotetramers produced by their constituent subunits. These findings have widespread implications for future signalling research and may help explain how specificity can be achieved by CNGCs that are known to act in disparate pathways. Regulation of complex formation may involve cyclic nucleotide-gated channel-like proteins.

## 1. Introduction

Plant cyclic nucleotide-gated channels (CNGCs) are held to be tetrameric cation channels formed by four subunits which may be activated by the cyclic nucleotide monophosphates (cNMPs) adenosine 3′,5′-cyclic monophosphate (cAMP) and guanosine 3′,5′-cyclic monophosphate (cGMP) [1,2,3,4,5,6,7,8,9,10]. The ability of cNMPs to act as signalling molecules in plants has been questioned. However, enhanced detection methods are revealing stimulus-induced increases and the ability to lower cAMP in cellular compartments using a “cAMP sponge” is now allowing the consequences of depletion to be investigated [11]. CNGCs are integral not only to plant nutrition, but also to calcium (Ca^2+^) signalling in development, abiotic stress and immunity [9,10,12,13,14,15]. Most research into CNGC contribution to signalling has focused on the 20 *cngc* loss-of-function mutants in *Arabidopsis thaliana* (summarised in Table 1) and on the functional characteristics of homotetrameric CNGCs in heterologous expression systems such as *Escherichia coli*, yeast, *Xenopus* oocytes and HEK293 cells. The 20 *AtCNGC* gene sequences [16] have been used to predict that *Glycine max* has 39 [17], *Hordeum vulgare* has 9 [7], *Nicotiana tabacum* has 35 [18], *Oryza sativa* has 16 [19], *Triticum aestivum* has 47 [20], *Zea mays* has 12 [21], *Brassica oleracea* has 26 [22] and *Brassica rapa* has 30 *CNGCs* [23]. Advances made with *Arabidopsis* may well have implications for crop species.

There is increasing evidence to suggest that CNGCs form heterotetrameric complexes which may have unique functional characteristics, compared to homotetrameric channels [9,10,27,28,29,44,46]. These may help facilitate the generation of stimulus-specific Ca^2+^ signatures (as monophasic, biphasic or oscillatory increases in this second messenger in a given cellular compartment) that could be decoded by specific complements of Ca^2+^-binding proteins to cause a stimulus-specific response [9,10]. These discoveries, in combination with recent advances in the model of CNGC structure, have major implications for our understanding of CNGC function and generate new areas for future research. Here, precedents for diverse channel subunit interactions are reviewed, with consideration of in vivo factors that may determine the subunits of CNGC complexes. Additionally, regulatory diversity of AtCNGC subunits is reviewed as a critical determinant of heterotetrameric channel function, with the possibility that CNGC-like proteins (if present) may restrict complex formation.

## 2. Heteromeric Channel Complexes Also Occur in Plants

It has been known for some time that animal cyclic nucleotide-gated (CNG) channels form heteromeric complexes and that the combinations of these subunits define the functional characteristics of the channel [68,69,70,71]. In mammalian retinal phototransduction, CNG channels in rod cells are formed by three subunits of CNGA1 and one CNGB1 subunit, with the C-terminal leucine zipper region of the CNGA1 subunits interacting to set the stoichiometry [70,71]. In contrast, in cone cells, the channel is formed by three subunits of CNGA3 and one CNGB3 subunit. It is held that the CNGB subunits “fine tune” channel behaviour by regulating opening/closing kinetics, affinity for cyclic nucleotides and ability to be regulated by Ca^2+^ [70]. Precedents for heterotetrameric channel assembly in plants have come from members of the Shaker voltage-dependent K^+^ channel family, underpinning K^+^ uptake and distribution. Interactions amongst Shaker channel subunits are dependent on specific regions of the cytosolic C-terminal domain (CT) and specific subunits may have inhibitory effects on the overall channel complex [72,73,74,75,76]. A breakthrough study on *Medicago truncatula* nodulation nuclear signalling revealed an interaction between the K^+^ channel DMI1 (Does not Make Infections 1) and MtCNGC15s, probably to enable voltage change mediated by DMI1 to promote MtCNGC15s opening and Ca^2+^ flux [77]. This opens up the possibility of interaction between entirely different channel families. Although there are no reports of interaction between K^+^ channels and CNGCs in *Arabidopsis* or other plants, there is now a good body of evidence that *Arabidopsis* CNGC subunits (AtCNGCs) also form heterotetrameric channels within the family. CNGC–CNGC interactions have also been proposed in *Zea mays* and the moss *Physcomitrella patens* [21,28].

**Figure 1:**
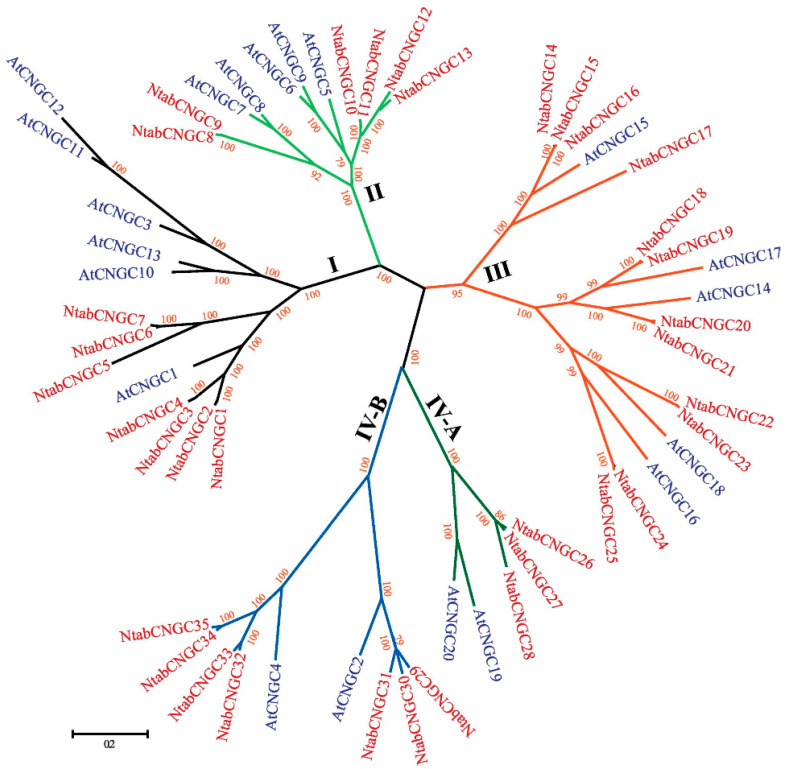
The phylogenetic relationship between CNGCs of *Arabidopsis thaliana* and *Nicotiana tabacum*. Multiple sequence alignment was carried out with the MUSCLE program and MEGA 6.0 used to generate the tree using the Jones–Taylor–Thornton (JTT) model. Bootstrap values from 1000 repeats are shown. Figure reproduced from Nawaz et al. 2019 [18]**,** with permission of the Publisher.

Bifluorescence complementation (BiFC) in *Nicotiana benthamiana* has shown interactions occur in planta between AtCNGC2 and AtCNGC4 [27], between AtCNGC8 and AtCNGC18 [46], between AtCNGC7 and AtCNGC18 [46], and between AtCNGC19 and AtCNGC20 [67]. BiFC analyses also suggest that AtCNGC6, AtCNGC9 and AtCNGC14 interact with each other (AtCNGC6/9, AtCNGC6/14, AtCNGC9/14) [44]. To demonstrate that BiFC signals are produced by heterotetrameric complexes, instead of clustered homotetramers, Pan et al. analysed single-molecule fluorescence to determine that co-expression of *AtCNGC7* or *AtCNGC8* with *AtCNGC18* resulted in heterotetrameric AtCNGC7/18 or AtCNGC8/18 complexes with a 2:2 stoichiometry [46]. This was an important breakthrough in establishing tetramer formation. Prior to this, the need for four CNGC subunits to combine to make a channel had been assumed by analogy with animal and bacterial channels, then supported by molecular modelling [78] (see Section 4 on channel structure). The range of subunit interactions found to date shows that they are not limited to co-members of the five phylogenetic groups within the family (I, II, III, IV-A and IV-B; Figure 1 [16,17,18,19,21,22,23,79]) but can occur across groups. Therefore, CNGC–CNGC interactions are isoform specific but are not restricted to closely related CNGC isoforms. In contrast, AtCNGC16 and AtCNGC18 interactions have not been observed [46], demonstrating that members of the same group (in this case group III) may not work together and that phylogeny alone may not be a useful tool in predicting interactions. Rather, a consideration of co-localisation is needed.

## 3. Complex Formation Would Depend on the Co-Localisation and Relative Abundance of CNGC Isoforms

Whether a CNGC is present in the plasma membrane or an endomembrane (as a homotetramer or in a heterotetrameric complex) has consequences for the generation of a signal-specific [Ca^2+^]_cyt_ signature. It is envisaged that CNGC plasma membrane localisation would play a part in signature initiation driven by receptors in that membrane whilst CNGCs in the tonoplast, for example, would be downstream and acting to amplify the [Ca^2+^]_cyt_ signal. Most CNGCs are believed to localise to the plasma membrane, and this has been reported for many AtCNGCs, including AtCNGC2 [33], AtCNGC3 [40], AtCNGC6 [44], AtCNGC7 [46], AtCNGC8 [46], AtCNGC9 [44], AtCNGC10 [48,80], AtCNGC11 [51], AtCNGC12 [51,81], AtCNGC14 [44], AtCNGC17 [60], AtCNGC18 [46,62,63], AtCNGC19 [66] and AtCNGC20 [82]. AtCNGC5 is reported to localise in microdomains at the periphery of *N. benthamiana* protoplasts when expressed heterologously [4]. It must be noted, however, that in contradiction of the findings by Meena et al. [66] and Fischer et al. [82], Yuen and Christopher reported that AtCNGC19 and AtCNGC20 localised to the vacuolar membrane, not the plasma membrane [83]. Similarly, Chang et al. found AtCNGC7, AtCNGC8 and AtCNGC16 to localise to endomembrane in pollen tubes, as opposed to the plasma membrane [61]. In silico predictions suggest that 11 of 12 *Zea mays* CNGCs could localise to the plasma membrane [21]. The importance of localisation is exemplified by the positioning of nuclear CNGCs to enable nuclear Ca^2+^ “spiking” in symbiosis signalling [77]. MtCNGC15a, MtCNGC15b and MtCNGC15c of *M. truncatula* localise to the nuclear envelope, with 14 of 21 MtCNGCs predicted to contain nuclear localisation sequences [77]. Recently, AtCNGC15 was also found to localise to the nuclear membrane and to be involved in root development [58]. From these results, it is clear that many CNGCs could co-localise, which would facilitate extensive CNGC–CNGC subunit interactions, including the formation of heterotetrameric complexes.

These interactions will necessarily be dependent on the co-expression of *CNGCs* within individual cells. Transcriptomic data indicate that many *CNGCs* (23 of 35 in *N. tabacum* [18]) are expressed throughout the plant. Expression levels, however, vary greatly, suggesting that many CNGCs have tissue-specific or cell-specific functions [4,18,21,22,23,44,47]. Expression of an individual CNGC can vary with development and growth conditions. For example, leaf expression of *AtCNGC3* increases with leaf age and lessens when the plant is grown in soil rather than on an agar plate [40]. *CNGC* promotors have been found to contain elements associated with responses to abscisic acid, auxin, ethylene, gibberellin, salicylic acid and methyl-jasmonate [17,18,19,20,21], and *CNGC* transcript levels are highly sensitive to abiotic and biotic stressors [17,18,19,20,22,23,79]. Notably, it has also been found that *Ziziphus jujube CNGC2* expression is rapidly induced by application of cAMP to callus [79], potentially providing a mechanism for priming signalling pathways involving ZjCNGC2. In silico analysis has also predicted that *NtabCNGC* expression may be regulated extensively by micro-RNAs and *cis*-acting regulatory elements [18]. Consequently, the abundance of different CNGC isoforms in each cell type is likely to be variable, and dependent on environmental conditions, which may result in the formation of different CNGC heterotetramers in different cell types.

A further consideration is that studies typically measure transcript levels from entire organs and may lack sufficient spatial resolution to detect low, cell-specific expression [17,18,19,22,23,44]. *AtCNGC5*, *AtCNGC6*, *AtCNGC9* and *AtCNGC14* have been implicated in root hair growth and their transcripts are abundant in roots [43,44,54,55,56,57]. However, *CNGC*promotor::GUS fusions of *AtCNGC6*, *AtCNGC9* and *AtCNGC14* also suggest that these *AtCNGCs* are expressed throughout the plant [44]. *AtCNGC6* expression is predicted in root, shoot, leaf and guard cells [4,44,45]. *AtCNGC9* expression is predicted in root hairs and guard cells but not leaves or shoots, and *AtCNGC14* expression is predicted in roots, shoots and the leaf [44]. As stated earlier, BiFC suggests that AtCNGC6, AtCNGC9 and AtCNGC14 may form a range of heterotetrameric complexes with each other [44]. From these data, it may be predicted that different complexes form in different cell types depending on the expression patterns of the interacting partners. Consequently, the signalling function of these AtCNGCs may be cell type specific. Notably, *AtCNGC6* has been implicated not only in root hair growth polarity but also in thermotolerance and cGMP-induced Ca^2+^ influx in guard cells (in which it could partner AtCNGC5) [4,28,44]. Additionally, whilst *AtCNGC9* has so far only been implicated in root hair growth polarity, its expression in guard cells would suggest a specific activity there as part of a complex with AtCNGC6. Both guard cell and mesophyll plasma membrane contain Ca^2+^ channels that are activated by cyclic nucleotides [84]. It is, therefore, possible that the formation of different CNGC complexes with unique functional characteristics in different cell types helps permit stimulus-specific signalling in the diverse pathways that AtCNGC6 and other AtCNGCs work in (Table 1).

The relative abundance of CNGC subunits is also likely to be a key determinant of CNGC complex formation and function. Yoshioka et al. discovered that the phenotypes associated with the *cpr22* mutant (a gene fusion between *AtCNGC11* and *AtCNGC12*) could be supressed by overexpression of *AtCNGC12*, and it was proposed that this was due to disruption of heterotetrameric complex formation [53]. Notably, *AtCNGC6* transcripts are >4.5 times more abundant than *AtCNGC9* transcripts in guard cells [4]. If these two CNGCs were to form heterotetrameric complexes (as suggested) and transcript abundance were proportional to protein abundance, then excess AtCNGC6 subunits must either form homotetramers, be degraded, sequestered in the membrane, or interact with additional AtCNGC subunits. Following a study of *AtCNGC2* homologues in *P. patens* by Finka et al., such an interaction between AtCNGC2 and AtCNGC6 has been proposed for heat signalling [28]. In pollen tube growth, the ratio of *AtCNGC18* to *AtCNGC8* expression is expected to determine the proportion of AtCNGC8/18 complexes relative to AtCNGC8 or AtCNGC18 homotetramers, leading to changes in cell permeability [46]. When expressed singly in *Xenopus* oocytes, AtCNCG18 forms a constitutively voltage-dependent, Ca^2+^-permeable channel but AtCNGC8 is electrically silent (as is AtCNGC7). Equimolar co-expression of *AtCNGC18* and *AtCNGC8* (presumably with heterotetramer formation) resulted in greatly reduced Ca^2+^ influx compared to *AtCNGC18* expression alone [46]. Pan et al. proposed that, by recruiting AtCNGC18 into heterotetrameric AtCNGC8/18 complexes, AtCNGC8 represses AtCNGC18 activity [46]. Consequently, whilst the overexpression of *AtCNGC18* in *A. thaliana* disrupts Ca^2+^-dependent pollen tube growth, potentially by forming deregulated AtCNGC18 homotetramers, this phenotype can be rescued by overexpressing *AtCNGC8*, presumably by recruiting AtCNGC18 subunits from homotetramers to generate heterotetramers [46]. However, it should be noted that Gao et al. reported that expression of *AtCNGC8* or *AtCNGC7* in HEK293T cells produced constitutively voltage-dependent, Ca^2+^-permeable channels which were activated further by addition of 8Br-cNMPs [5]. This contradiction may be caused by the different bathing solutions used in each study or the use of different heterologous expression systems. Examples of key findings from transport studies on heterologously expressed CNGC genes are shown in Table 2. This further shows that the choice of expression system may have an effect on the outcome. For example, K^+^ selectivity over Na^+^ of AtCNGC2 was greater in HEK293 cells than in *Xenopus* oocytes [2]. Nevertheless, AtCNGC7/18, and AtCNGC8/18 complexes appear to form spontaneously in a heterologous expression system, yielding different transport characteristics to the homomeric forms [46]. If single-molecule fluorescence were to confirm the existence of an AtCNGC2/4 complex, as well as complexes amongst the AtCNGC6, AtCNGC9 and AtCNGC14 triad and AtCNGC19/20 couple as predicted by BiFC [27,44,67], then it is likely that CNGC complexes could be widespread if co-localisation permits.

## 4. CNGCs Are Extensively Regulated—Formation of CNGC Complexes Generates Further Regulatory and Functional Complexity

**Figure 2:**
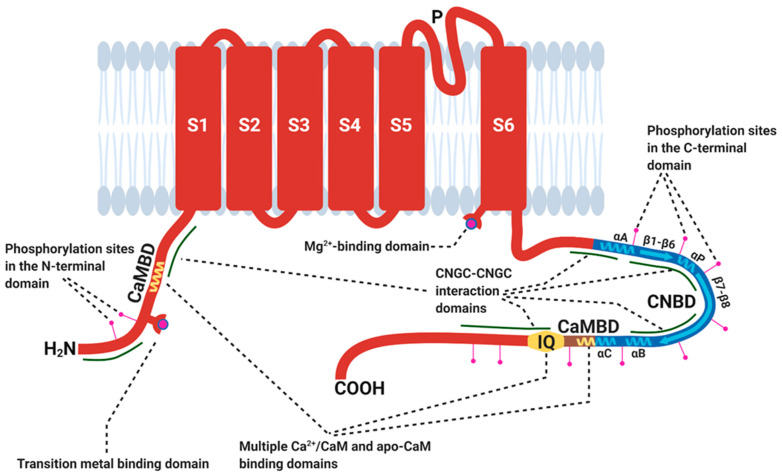
Cyclic nucleotide-gated channel (CNGC) subunit structure. Plant CNGCs consist of six transmembrane domains (S1–S6) with a pore region between S5 and S6. Both N- and C-terminal domains are cytosolic. In the C-terminal domain is a cyclic nucleotide-binding domain (CNBD), which is believed to be formed of four α-helices (αA, αP, αB, αC) and eight β-sheets (β1–β8). Overlapping with the C-terminal side of the CNBD is a calmodulin (CaM)-binding domain (CaMBD). CNGCs also contain a CaM-binding IQ (isoleucine-glutamine) motif. From the data presented by Pan et al. 2019 [46] and Chiasson et al. 2017 [99], the N- and C-terminal regions are predicted to contain CNGC–CNGC interaction domains. Lemtiri-Chlieh et al. 2020 [96] predict the presence of a Mg^2+^-binding domain downstream of the CNGC pore. Demidchik et al. 2014 [99] predict a transition metal-binding domain in the N-terminus of AtCNGC19 and AtCNGC20. CNGC phosphorylation is likely for AtCNGC4 [29], AtCNGC18 [63], AtCNGC19 [67], AtCNGC20 [67] and OsCNGC9 [89]. An N-terminal CaMBD has been identified in AtCNGC12 [50]. Not all plant CNGCs contain all the structures displayed on this image. Structure was adapted from Chin et al. 2009 [12] and Kaplan et al. 2007 [101]. Figure created with BioRender.com.

Here we summarise the current understanding of CNGC structure and regulation, discussing how the formation of CNGC complexes may further affect CNGC regulation and function. The breakthrough studies on plant CNGCs used primary structures of potassium channels and animal CNG subunits to assign domains [91,92,93]. The overall model CNGC subunit has six transmembrane domains (S1–S6; Figure 2) with a pore region (P loop) between S5 and S6 that permits ion transport [13]. Animal and bacterial cation channel subunits that contain a single P loop form tetramers; this includes animal CNGs, with clear evidence from cryo-electron microscopy showing tetramer formation in a lipid environment [94]. Triplet amino acid residues in the P loop that could act as selectivity filters (AGN, AND, GNL, GQG, GQN, GQS) vary between the *Arabidopsis* CNGCs [95], with AND or GQs thought to confer some level of Ca^2+^ selectivity [4,5,6,30,35,45,78]. Recent analysis of AtCNGCs has revealed the presence of a diacidic motif for Mg^2+^ binding close to the pore region in the cytosolic CT in all but AtCNGC2. It has been proposed that this could account for channel blocking by cytosolic Mg^2+^; the consequences for signalling and nutrition have yet to be explored [96]. The CT contains a cyclic nucleotide-binding domain (CNBD) which is believed to be formed of four α-helices (αA, αP, αB, αC) and eight β-sheets (β1–β8). Overlapping with the C-terminal side of the CNBD is a calmodulin (CaM)-binding domain (CaMBD) [97,98]. A CaM-binding IQ (isoleucine-glutamine) motif is also present [18,21,23,82] and the CT can contain multiple phosphorylation sites [29,67,89]. The CT of AtCNGC8 appears necessary and sufficient for interactions between AtCNGC8 and AtCNGC18 subunits in *Xenopus* oocytes [46]. There is also a short, cytosolic N-terminal domain (NT) that is predicted to harbour CNGC–CNGC interaction domains [99] and may contain phosphorylation sites [29,67]. The NT of AtCNGC12 contains a CaMBD [50]. The AtCNGC19 and AtCNGC20 NTs are predicted to have cysteine residues that could form an Fe/Cu-binding site to act as a Fenton catalyst in the production of hydroxyl radicals regulate plant Ca^2+^ channel activity in growth and stress responses [99,100].

### 4.1. CNGCs Are Regulated by cNMPs That May Be Generated by Soluble or Membrane Proteins

cAMP and cGMP are secondary messengers which are synthesised by adenylyl cyclases (ACs) and guanylyl cyclases (GCs), respectively. cNMP gating of CNGCs is well documented and is summarised in Table 2, but historically the physiological importance of cNMPs has been controversial [11,102,103,104]. cNMP levels in plants are significantly lower than in animals and, until the development of more sensitive assays, it was doubted whether cNMPs were present at all [102]. To date, cAMP has been implicated in seed germination and cell cycle progression, pollen tube growth and orientation, stomatal kinetics, photosynthesis and photorespiration, abiotic stress responses (heat and chill stress, salinity, drought, aluminium, nutrient deficiency), wounding and immunity [11]. A range of soluble and membrane proteins has now been identified with potential AC or GC activity, in their cytosolic domains for the membrane proteins. For ACs, these include the K^+^ uptake transporters AtKUP5 and AtKUP7 (K^+^ uptake permease) [105,106], and AtLRRAC1 (leucine-rich repeat adenylyl cyclase1) [107]. For GCs, AtGC1 (guanylyl cyclase1) [108], AtNOGC1 (nitric oxide-dependent guanylate cyclase1) [109], AtPSKR1 (phytosulfokine receptor1) [110], AtPepR1 (plant elicitor peptide receptor1) [3], AtBRI1 (brassinosteroid insenstive1) [111] and AtWAKL10 (wall-associated kinase (WAK)-like10) [112] have all been identified and studied in vitro. Two homologues of AtPepR1 have now been identified in tomato (*Solanum lycopersicum* L.; SlGC17, SlGC18) and have been reported to have GC activity that is required for [Ca^2+^]_cyt_ increase in response to flg22, chitin and AtPep1 [113]. Analysis of recombinant protein activity suggests that AC and GC activity in plants is typically much lower than in animal counterparts, with pmol or fmol cNMP μg^−1^ protein min^−1^ values reported [3,107,108,109,111]. However, the membrane-bound AtWAKL10 and AtPSKR1 have a *V_max_* of approximately 2 µmol mg^−1^ min^−1^ [110,112]. It is possible that, in planta, CNGC cNMP sensitivity is increased by CaM, phosphorylation, or formation of heterotetrameric complexes, and so lower cNMP concentrations are required than those used in heterologous expression systems or native membranes in transport studies.

It may be that the low activity of plant ACs and GCs is central to signal specificity. If the domains were in close proximity to specific CNGCs, generating cNMP concentrations sufficient to activate those channels [3], then crosstalk between separate CNGC-dependent signalling pathways could be eliminated—only CNGCs co-localising with the AC or GC would be activated. How close is close enough? At the plasma membrane, both AtCNGC17 and AtPSKR1 have been found to interact with AtBAK1 (BRI-associated receptor kinase1) and although AtCNGC17 does not interact with the PSKR1 receptor that generates cGMP, this channel is essential for phytosulfokine/PSKR1-dependent protoplast expansion involving the H^+^-ATPases AtAHA1 and AtAHA2 and is thought to form part of this multi-protein complex [60]. Increased [Ca^2+^]_cyt_ promotes PSKR1’s GC activity but inhibits its kinase activity [114], raising the possibility that AtCNGC17-mediated [Ca^2+^]_cyt_ elevation not only generates a positive feedback loop for the cGMP pathway but could also curtail any phosphorylation-dependent pathway to ensure signalling specificity. A positive feedback loop may also explain the jasmonic acid-induced rise in cAMP in leaf epidermal cells that requires AtCNGC2 [35]. It is possible that AtCNGC2-mediated Ca^2+^ influx activates ACs either directly or via intermediates such as calcium-dependent protein kinases and CaMs.

The recent finding that *Arabidopsis* root hair K^+^ influx precedes increased growth rate and can cause [Ca^2+^]_cyt_ increase [115] begs the question of whether AC activity of AtKUP5 and AtKUP7 is involved. Both these K^+^ transporters are expressed in root hairs [116], and AtKUP7 is in the plasma membrane [117]. When expressed in yeast, AtKUP5-mediated K^+^ influx causes cAMP accumulation [106]. This suggests a model in which KUP-mediated K^+^ influx to the root hair causes cAMP increase to activate the CNGCs (AtCNGC5,6,9,14) implicated in Ca^2+^ influx and polar growth [43,44,55,56,57]. As AtCNGC5, AtCNGC6 and AtCNGC9 appear to transport Ca^2+^ rather than monovalent cations [4,6,43] (Table 2), it seems likely these subunits are relevant to Ca^2+^ signalling. The spatial localisation of the KUPs relative to the CNGCs is worthy of attention. Activation of CNGCs with strong K^+^ permeation could conceivably contribute to K^+^ uptake in root hairs and other cells, indeed AtCNGC3 and AtCNGC10 are held to be important for root K^+^ acquisition [48,101].

Salt stress causes cGMP accumulation within seconds in *Arabidopsis* seedlings [118] and CNGCs have been proposed to be part of the salt-induced [Ca^2+^]_cyt_ signalling response [119]. In *Arabidopsis*, cNMPs can restrict Na^+^ influx rather than promote it, ostensibly by reducing the open probability of root plasma membrane Na^+^-permeable channels (an effect observed in approximately half of the patch clamp trials) [120]. This would imply negative regulation by cNMPs of a putative CNGC channel. Notably, AtCNGC3 and AtCNGC10 appear to contribute to Na^+^ uptake [40,49]. Patch clamp electrophysiological analysis of *Arabidopsis* root epidermal plasma membrane has also revealed a Na^+^ influx channel that could not discriminate against K^+^ [121] (a “non-selective” cation channel [122]) and this was proposed to be Ca^2+^ permeable in a later study [123]. It is not known whether the channel is regulated by cNMPs and could account for the negative effects of cNMPs on Na^+^ influx reported by Maathuis and Sanders, 2001 [120]. The roles of cNMPs and CNGCs in salt stress urgently require further elucidation. Understanding which CNGC subunits and potential heteromeric complexes are expressed in different root cells (which vary in their salt-induced [Ca^2+^]_cyt_ response [124]), what their functional permeability is to Na^+^ and Ca^2+^ and how they are regulated by cNMPs is likely to be of great importance.

### 4.2. CNGCs Are Positively and Negatively Regulated by CaM, Potentially Affording Ca^2+^ Sensing and Feedback

Calmodulins are Ca^2+^-binding proteins with a major role in Ca^2+^ signal transduction in plants [10,29,46]. In former models of CNGC activity, CaM was believed to have an exclusively inhibitory effect where the Ca^2+^-bound form of CaM (Ca^2+^/CaM) inhibited CNGCs by perturbing cNMP gating, competing for a binding site internal to the CNBD [98]. Subsequently, multiple CaMBDs have been identified, with structural divergence amongst CNGC isoforms. For example, AtCNGC12 contains an N-terminal CaMBD which interacts with Ca^2+^/CaM and could result in channel closure [50]. In addition, an IQ domain has been identified which is C-terminal to the CNBD and conserved in the majority of plant CNGCs [18,21,23,85]. Yeast 2-hybrid assays suggest that interactions between CNGC CTs and CaM isoforms are specific, with the IQ domain contributing to many of these interactions [33]. Indeed, CaM isoform-specific effects are now being documented [10], for example root hair AtCNGC14 is negatively regulated by AtCaM7 binding to its CT but not by AtCaM2 [56]. Additionally, AtCNGC6 is inhibited by AtCaM2,3,5,7 at the IQ domain in heat shock signalling but not by AtCaM1,4 or 6 [125].

It has been proposed that apo-CaM (CaM without Ca^2+^ ligands) constitutively binds to the IQ domain in a Ca^2+^-independent manner to act as a Ca^2+^ sensor [10,33,50]. The model arising from studies on AtCNGC12 has the channel’s opening causing local Ca^2+^ elevation, hence permitting Ca^2+^ binding to apo-CaM [50]. That initial channel opening could be triggered by membrane hyperpolarisation because when expressed in *Xenopus*, AtCNGC12 presents as a hyperpolarisation-activated Ca^2+^ channel that does not require cNMPs [8]. Ca^2+^-CaM interaction at the IQ domains of adjacent subunits and Ca^2+^-CaM recruitment to CaMBDs could modulate channel activity [50]. Evidence from *Xenopus* expression points to AtCaM1 as an activating ligand [8]. As Ca^2+^ increases, Ca^2+^-CaM binding to the NT CaMBD effects channel closure [10,50]. Much, therefore, depends on which CaM isoforms are locally available and their affinities for Ca^2+^ and the CaMBDs. A further model built on AtCNGC8/18 activity in *Xenopus* coupled with analysis of CT binding has apo-CaM2 binding to the IQ domains to counter the inhibitory effect of AtCNGC8 and so promote channel opening. As Ca^2+^ increases as a consequence, Ca^2+^-CaM2 forms but then dissociates to promote channel closure [46]. Expressing *AtCNGC8* and *AtCNGC18* with *AtCaM2* in HEK293 cells leads to [Ca^2+^]_cyt_ oscillations [46], which has implications for pollen tube apical [Ca^2+^]_cyt_ oscillations during growth. It remains to be seen how cNMPs fit into this regulatory complex, given activation of AtCNGC18 by cGMP in native pollen plasma membrane [6] and by cNMPs in heterologous expression [5,63].

CaM regulation of CNGCs may be important in immune signalling. BIK1 is a receptor-like cytoplasmic kinase which acts downstream of FLS2 (Flagellin Sensitive2) [126], an LRR receptor-like kinase which binds to the bacterial flg22 peptide and is required for [Ca^2+^]_cyt_ elevation [127]. Although at the whole-plant level (which may lack sufficient resolution) AtCNGC2 was reported to have no involvement in flg22-induced [Ca^2+^]_cyt_ increase [128], a genetic analysis of *AtCNGC2* and *AtCNGC4* concluded that both genes act in the flg22 pathway [27]. At the leaf disc level, use of loss-of-function mutants indicated that both AtCNGC2 and AtCNGC4 are involved in flg22-induced [Ca^2+^]_cyt_ increase, given a permissive apoplastic Ca^2+^ level [29]. Similarly, patch clamping of mesophyll protoplasts showed that both were needed for flg22-induced plasma membrane Ca^2+^ influx currents [29]. It should be noted, however, that flg22-induced plasma membrane depolarisation of individual mesophyll cells (which involves Ca^2+^ influx) was found to be independent of *AtCNGC2* [129]. Following the results of heterologous co-expression in *Xenopus* oocytes [29], it is likely the subunits form a AtCNGC2/4 complex. Although single expression of either *AtCNGC2* or *AtCNGC4* in *Xenopus* oocytes failed to cause channel activity, their co-expression produced a hyperpolarisation-activated Ca^2+^-permeable channel that did not require cNMPs [29]. This channel activity could be supressed by the co-expression of *AtCAM7* and this suppression could be overcome by the additional expression of *AtBIK1* [29]. It was subsequently found that application of flg22 induces AtBIK1-mediated phosphorylation of the AtCNGC4-CT in planta, which is believed to overcome AtCaM7-mediated repression [9,29]. More recently, a split luciferase complementation assay using nano-luciferase suggests that the CT and NT of homomeric AtCNGC2 and AtCNGC4 may move apart when challenged with flg22 in planta [130]. It may be postulated that this change is linked to the disassembly of homomeric complexes and the formation of heterotetrameric complexes. Facultative complex formation may contribute to the ability for CNGC subunits to carry out multiple signalling roles.

Again, it remains to be seen what role, if any, cNMPs play in this pathway. Electrophysiological analyses support cNMP activation of AtCNGC2 either in native membrane or when heterologously expressed (Table 2; [30,38]) and with an apparent ability to discriminate between cAMP and cGMP in guard cells [4,30]. As flg22 can induce guard cell [Ca^2+^]_cyt_ oscillations [131] and as CNGCs could be involved in [Ca^2+^]_cyt_ oscillations [46], further consideration of guard cell AtCNGC2 in this immune pathway is warranted. The recent discovery that BIK1 phosphorylates the guard cell plasma membrane Ca^2+^ channel AtOSCA1.3 (hyperOsmolality-induced [Ca^2+^]i increase 1.3) as part of the stomatal flg22 response [132] still leaves room for other Ca^2+^ influx pathways. Moving away from *Arabidopsis*, SlCNGC1 and SlCNGC14 are required for the tomato flg22-induced [Ca^2+^]_cyt_ increase but it is not yet clear whether these subunits can form a complex [133]. Overall, the study of CNGC regulation by CaM is complicated by the abundance of CaM and CNGC isoforms and the multitude of CaMBDs. It is likely that this complexity contributes to the specificity of signal transduction by CNGCs.

### 4.3. CNGCs Are Regulated by Phosphorylation

CNGC phosphorylation is emerging as an important regulator of activity. AtCNGC4 contains nine phosphorylation sites within and around the CT CNBD and, as described in Section 4.2, phosphorylation by BIK1 relieves CaM7-mediated inhibition of the putative AtCNGC2/4 complex in flg22 signalling [29]. Similarly, the rice receptor-like cytoplasmic kinase OsRLCK185 (receptor-like cytoplasmic kinase 185) is responsible for activation of OsCNGC9 by phosphorylation, triggering defence responses [89]. *AtCNGC19* and *AtCNGC20* have also been found to play a role in plant defence downstream of BAK1/SERK4 (somatic embryogenesis receptor kinase 4). However, in this case, the authors proposed that the signalling cascade progressed through BAK1-mediated phosphorylation of the AtCNGC20-CT, leading to proteasome-dependent degradation, as opposed to phosphorylation-mediated channel activation [67]. Mutation of Thr^560^/Ser^617^/Ser^618^/Thr^619^ in the AtCNGC20-CT reduced BAK1-mediated phosphorylation, and additional phosphorylation sites were predicted in the C- and N-terminals [67]. It was not reported, however, whether AtCNGC20-CT phosphorylation might also be an activating signal, which may subsequently be followed by signal termination via protein degradation. It is possible, therefore, that AtCNGC20-CT phosphorylation may overcome CaM-mediated inhibition, as found with AtCNGC2/4 in the flg22 pathway. It is also possible that AtCNGC2/4 phosphorylation may promote proteasome-mediated degradation and, as such, CNGC phosphorylation may have dual function in planta.

The role of CNGC phosphorylation is not restricted to defence signalling. Calcium-dependent protein kinase 32 (CPK32) appears to interact with AtCNGC18 in planta, increases the conductance of AtCNGC18 homotetramers when co-expressed in *Xenopus* oocytes and, following overexpression in pollen tubes, leads to increased apical [Ca^2+^]_cyt_ [63]. Following the identification of AtCNGC8/18 heterotetramers, it would be interesting to test how CPK32 affects AtCNGC8/18 activity. In silico analysis also predicts numerous phosphorylation sites in CNGCs from *N. tabacum* [18] and *Brassica oleracea* [22], suggesting that kinase/phosphatase regulation of CNGC activity is widespread.

### 4.4. CNGC Complexes Generate Further Functional and Regulatory Complexity

As discussed, the functional characteristics of heterologously expressed AtCNGC8/18 and the putative AtCNGC2/4 complex can be different from the homotetrameric channels of their constituent subunits, including changes to cNMP gating [29,46]. It also appears that CNGC complexes display changes in ion selectivity. AtCNGC2 can conduct K^+^, Cs^+^, Rb^+^ and Li^+^ but has a very low permeability to Na^+^ which correlates with a change in pore region amino acid sequence, from GQN to AND [85]. It would appear, therefore, that AtCNGC2 has a role in mineral nutrition where Na^+^ permeability is particularly deleterious. AtCNGC2 has been implicated in uptake of Ca^2+^ into leaves [38] and it may be that Na^+^ exclusion is important in this function. Unlike AtCNGC2, AtCNGC4 appears to be permeable to both K^+^ and Na^+^ [41]. However, co-expression of *AtCNGC2* and *AtCNGC4* forms channels which are permeable to K^+^ but impermeable to Na^+^ [29]. This supports the hypothesis that AtCNGC2 and AtCNGC4 do form complexes and suggests that these complexes have unique functional characteristics, including permeability. It is important for future studies of CNGC permeability, therefore, to consider whether the CNGCs being tested exist as complexes in planta. For example, Zhang et al. reported that expression of *AtCNGC14* in *Xenopus* oocytes produced channels which were permeable to Mg^2+^ but effectively impermeable to K^+^, Na^+^ and Ba^2+^, under the conditions tested [57]. It should be investigated whether AtCNGC complexes containing AtCNGC14 also display selectivity against these ions.

**Figure 3:**
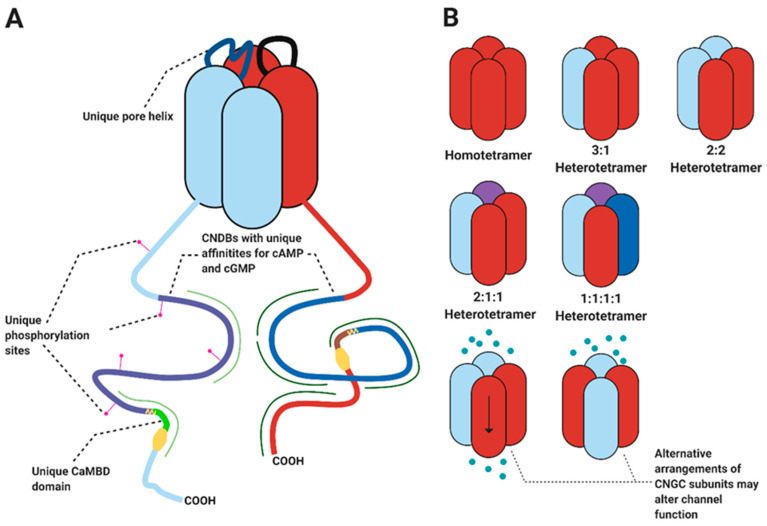
(**A**) Heterotetrameric complexes possess non-identical C-terminal domains. Differences in the cyclic nucleotide binding domain may determine specificity for cGMP or cAMP. Different CaM isoforms show preferential binding with different CNGC C-terminal domains [8,33], so heterotetrameric complexes may show combinatorial control by different CaM isoforms. Different CNGC C-terminal domains will be phosphorylation targets of different protein kinases, providing a mechanism for signal integration. The combination of different pore helixes may lead to changes in CNGC ion selectivity. For simplicity not all pore helices or cytosolic domains are shown. (**B**) Multiple different arrangements of CNGC subunits could be possible. CNGC activity may be regulated by both the different isoforms involved and their arrangement. Figure created with BioRender.com.

CNGC complexes will also make allosteric regulation of CNGC activity more intricate. For example, since CNGC complexes will be composed of multiple CNGC isoforms, with non-identical CTs and NTs (Figure 3A), the CNGC complex may interact with new combinations of allosteric regulators, including different CaM isoforms or different protein kinases. Each combination is likely to be unique to each CNGC complex and could result in unique feedback loops. Consequently, CNGC complexes may produce characteristic Ca^2+^ signatures which would enable CNGC complexes, even those that share a CNGC subunit, to participate in different signalling pathways. In addition, it is possible that the selectivity of some CNGCs, such as HvCNGC2-3 [7], for one of cAMP or cGMP may lead to specificity in heterotetrameric complexes.

An additional consideration is how CNGC subunits may compete for interactions with other CNGC isoforms. For example, it is apparent that AtCNGC7 and AtCNGC8 preferentially interact with AtCNGC18 in *Xenopus* to form AtCNGC7/18 or AtCNGC8/18 heterotetrameric complexes, instead of forming homotetrameric complexes [46]. It is also likely that AtCNGC2 and AtCNGC4 also preferentially interact to form AtCNGC2/4 heterotetramers, as opposed to homotetramers. Therefore, in a situation where two or more possible CNGC complexes may be formed, it is likely that particular CNGC complexes will form preferentially over others. Understanding these interaction dynamics may be key to understanding CNGC activity. The *brush* mutation in *Lotus japonicus* is an exemplar of how small changes in CNGC structure can significantly alter CNGC complex function in planta [99]. *BRUSH* is an *LjCNGC* homologous to AtCNGC19 and AtCNGC20 [99]. The *brush* mutation is found within the CNGC N-terminus and leads to a quantitative gain-of-function phenotype associated with the constitutive, voltage-dependent Ca^2+^ permeability of the *brush* homotetramer [99]. Competition between alternative CNGC subunits is believed to restrict formation of this homotetramer except in those plants strongly expressing *brush* [99].

There also remain a number of avenues which have remained unexplored in the study of plant CNGC complexes. For example, it remains unknown whether plant CNGCs form complexes with three or four different subunits (Figure 3B). Furthermore, whilst it has been assumed in models of CNGC complexes that the stoichiometry of CNGC subunits is 2:2 [10,67], there is only evidence supporting that assumption in the case of AtCNGC8/18 and AtCNGC7/18 [46]. It is also possible that CNGC function may be altered by the order in which CNGC subunits are ordered around the channel pore. In their analysis of animal CNG channels, Liu et al. discovered that the order of CNG subunits could alter channel conductance by up to 50% [69]. Two CNGC complexes, therefore, whilst having the same stoichiometry of CNGC subunits, may display different functional characteristics. Perhaps the formation of CNGC complexes, and the order of CNGC subunits, in planta is influenced by allosteric regulators such as CaM, which may promote stronger interactions between different CNGC subunits, and help dictate their order. Alternatively, it is possible that where the order of CNGC subunits differs, the conductance, ion selectivity and interactions with allosteric regulators are all altered, leading to divergent functional outcomes between otherwise similar CNGC complexes.

In silico analysis of CNGC sequences have identified several uncharacterised motifs which may further enhance CNGC regulation and function. Nawaz et al. identified three uncharacterised NtabCNGC motifs that are approximately 50 amino acids long [18], as well as a raffinose synthase motif in all 35 NtabCNGCs [18]. Furthermore, three uncharacterised motifs in *Brassica rapa* CNGCs are found in a number of closely related BraCNGCs, suggesting that these BraCNGCs have additional functionality [23]. It is possible that some of these uncharacterised motifs contribute to CNGC subunit interactions and, if heterotetrameric complexes were confirmed to be widespread amongst CNGCs, these complexes would significantly increase the complexity of CNGC regulation.

## 5. Could CNGCLs Modulate Complex Formation?

**Figure 4:**
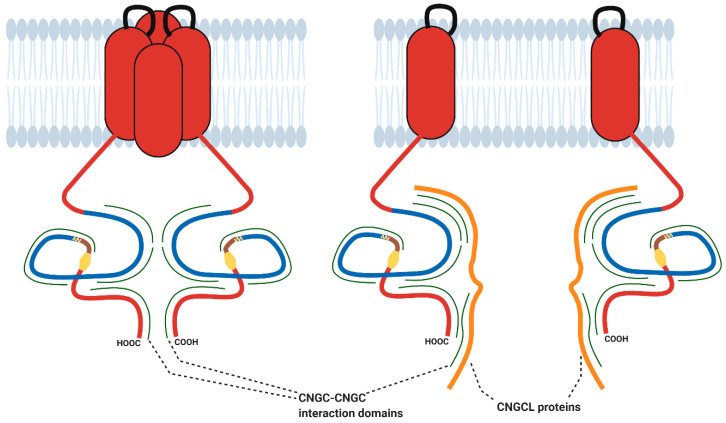
CNGCL proteins may disrupt the interactions between CNGC subunits and destabilise CNGC tetramers. For simplicity, pore loops and CT are shown in only two subunits. Figure created with BioRender.com.

Angiosperm evolution has seen the loss of several types of Ca^2+^ channel that are still found in animals and an apparent overall reduction in diversity of Ca^2+^ influx mechanisms compared to animals [134]. This suggests a greater reliance on the channels that were retained over evolution such as CNGCs. By comparison, many plant species harbour more cyclic nucleotide-gated channel subunits than animals. Vertebrates (including mammals) and invertebrates have only six CNGs [94,98,135]. Subunits of the hyperpolarisation activated cyclic nucleotide-gated (HCN) cation channels (that operate in cardiac cells) are also present in low numbers (three in invertebrates, four in mammals and four to six in other vertebrates) [136]. An ability to form diverse CNGC complexes from a greater number of subunits could compensate for the reduction in diversity of Ca^2+^ influx mechanism evident in plant genomes and fit each cell to respond appropriately to the diverse and coincident stimuli experienced during their sessile lives. Additionally, a range of homomeric or heteromeric CNGC channels could permit function beyond Ca^2+^ signalling and help explain the role of CNGCs in mineral nutrition. Truncated, CNGC-like (CNGCL) proteins may also provide a further layer of regulation by modulating CNGC complex formation (Figure 4). Pan et al. demonstrated that the AtCNGC8 CT inhibits AtCNGC18 activity [46]. This is likely due to the formation of AtCNGC8/18 heterotetramer-like interactions which prevent formation of the AtCNGC18 homotetramer. In principle, therefore, any CNGC CT could disrupt CNGC–CNGC interactions. Likewise, since the data presented by Chiasson et al. suggest that the CNGC NT also contains CNGC–CNGC interaction domains [137], it is possible that any CNGC NT could also disrupt CNGC–CNGC interactions. Genome-wide analysis of *CNGC* sequences has identified a number of truncated *CNGC* genes in *B. rapa*, *B. oleracea* and *N. tabacum* which were not analysed further in the original studies since they lack key CNGC domains [18,21,22].

**Figure A1:**
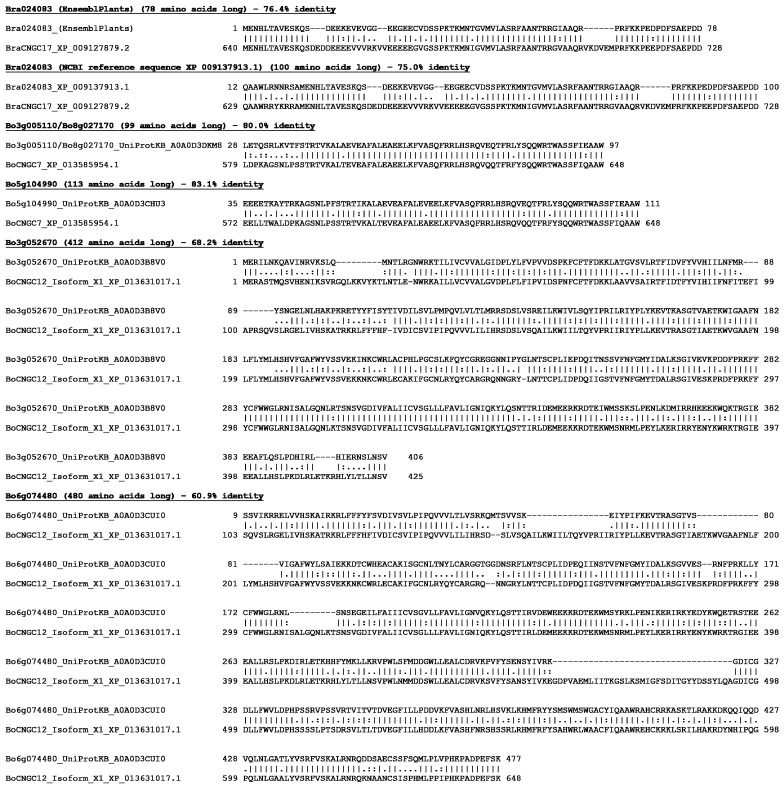
Local sequence alignments of CNGCL protein sequences against CNGC protein sequences. In each case the CNGCL sequence is displayed above the CNGC sequence. In each subheading the full length of the CNGCL sequence is given as well as the percentage identity with the CNGC sequence shown.

We examined some of these CNGCL sequences in silico to determine whether they may have the potential to disrupt CNGC–CNGC interactions. Six CNGCL genes (*Bra024083*, *Bo3g005110*, *Bo8g027170*, *Bo5g104990*, *Bo3g052670* and *Bo6g074480*) were identified from genome-wide analyses of *B. rapa* and *B. oleracea* and located in the EnsemblPlants database (https://plants.ensembl.org/index.html) [22,23]. For the five *B. oleracea* genes, the protein sequences were extracted from their UniProtKB identifiers. For *Bra024083*, the annotation in EnsemblPlants predicts a 78 amino acid sequence, whereas the NCBI reference sequence for *Bra024083*, XP_009137913.1, predicts a 100 amino acid protein. Both sequences were used in the subsequent analysis. The seven protein sequences were used as queries to search for homologous sequences in the genomes of *B. rapa* and *B. oleracea* using the NCBI BLAST protein program with default parameters (https://blast.ncbi.nlm.nih.gov/Blast.cgi?PAGE=Proteins). Following this, protein sequences of CNGCLs and the most similar CNGC identified in the BLAST search were submitted to pairwise local sequence using the EMBOSS Water program with default parameters (https://www.ebi.ac.uk/Tools/psa/emboss_water/). These alignments are presented in Figure A1 in Appendix A.

*Bra024083* (NCBI reference sequence XP_009137913.1) was initially identified for its homology with *AtCNGC17* [23]. In our analysis, the 78 amino acid prediction aligns to a region in the CT of BraCNGC17 (NCBI reference sequence XP_009127879.2, positions 640–728) with 76.4% identity. The 100 amino acid prediction aligns to an overlapping region of BraCNGC17 (positions 629–728) with 75% identity. In *B. oleracea*, *Bo3g005110* (UniProtKB_A0A0D3DKM8) and *Bo8g027170* (UniProtKB_A0A0D3DKM8) are predicted to encode identical 99 amino acid peptides which align to a 70 amino acid stretch in the CT of BoCNGC7 (NCBI reference sequence XP_013585954.1, positions 579–648) with 80.0% identity. Similarly, *Bo5g104990* (UniProtKB_A0A0D3CHU3) is predicted to encode a 113 amino acid peptide which aligns to a 76 amino acid stretch in the CT of BoCNGC7 (positions 572–648) with 83.1% identity. *Bo3g052670* (UniProtKB_A0A0D3B8V0) is predicted to encode a 412 amino acid peptide which aligns to the NT sequence of BoCNGC12 (NCBI reference sequence XP_013631017.1, positions 1–425) with 68.2% identity and *Bo6g074480* is predicted to encode a 480 amino acid peptide which aligns with BoCNGC12 (positions 103–648) with 60.9% identity. In silico observations may be misleading but these putative CNGCL proteins may warrant further attention to determine whether they are functional in planta and interact with CNGCs.

## 6. Conclusions

Research into plant CNGCs has historically focused on the role of individual *CNGC* genes. There is increasing evidence, however, to suggest that plant CNGCs function as heterotetrameric complexes.

To understand the role of CNGCs (whether in Ca^2+^ signalling or nutrition), it is necessary to determine which subunits interact and determine whether they form heterotetrameric complexes. If CNGC complexes were common, it will be important to determine which CNGC interactions occur preferentially and to study *CNGC* expression patterns to help predict the composition of CNGC complexes in different cell types. Consequently, a systematic study of CNGC interactions through BiFC and single-molecule fluorescence is needed to understand which complexes may be present in planta. In common with animal studies, cryo-electron microscopy should be able to resolve tetrameric structures. The advent of fluorophore-labelled cyclic nucleotides is now enabling the effect of cNMP binding on channel kinetics to be elucidated for animal homomeric and heterotetrameric CNGs [138] and could be applied to heterologously expressed plant CNGCs to further understand differences between complexes. It will subsequently be important to test the activities of these CNGCs in physiologically relevant conditions and determine how their behaviour is different from homotetrameric channels.

Recent studies, therefore, have significant implications for the future of CNGC research and may herald a major shift in our understanding of CNGC function. The role of CNGCs in plants may, truly, be complex.

## Figures and Tables

**Table 1:** *AtCNGCs* are involved in diverse signalling pathways ranging from development to stress responses. For each CNGC in *Arabidopsis thaliana*, the reported physiological or developmental roles are presented, based on phenotyping loss-of-function mutants. Where the roles of two or more CNGCs overlap, it may be postulated that a CNGC complex might form between these subunits if they co-localise. Currently, complex formation has only been investigated in a few of these cases.

Gene	Proposed Physiological or Developmental Process	References
*AtCNGC1*	Negative regulation of Pb^2+^ tolerance; primary root growth; gravitropism	[24,25,26]
*AtCNGC2*	Pathogen defence; programmed cell death; nitric oxide generation; suppression of leaf senescence; flowering time; thermotolerance (heat and chill); Ca^2+^ transport in leaves and Ca^2+^ sensitivity; jasmonic acid-induced Ca^2+^ entry	[27,28,29,30,31,32,33,34,35,36,37,38,39]
*AtCNGC3*	Germination; salt tolerance; Na^+^ and K^+^ uptake	[40]
*AtCNGC4*	Pathogen defence; programmed cell death; flowering time; thermotolerance (heat and chill); Ca^2+^ tolerance	[27,28,29,32,38,41]
*AtCNGC5*	cGMP-activated Ca^2+^ entry in guard cells; salt tolerance; root hair growth; auxin signalling	[4,42,43]
*AtCNGC6*	cGMP-activated Ca^2+^ entry in guard cells; thermotolerance (heat); root hair growth; auxin signalling	[4,43,44,45]
*AtCNGC7*	Pollen tube growth	[46,47]
*AtCNGC8*	Pollen tube growth	[46,47]
*AtCNGC9*	Root hair growth; auxin signalling	[43,44]
*AtCNGC10*	Negative regulation of salt tolerance; K^+^, Na^+^ and Pb^2+^ uptake; K^+^ homeostasis; negative regulation of Pb^2+^ tolerance; regulation of starch granule size; gravitropism; flowering time; hypocotyl elongation	[25,48,49]
*AtCNGC11*	Pathogen defence; programmed cell death; Pb^2+^ and Cd^2+^ uptake; Pb^2+^ tolerance; negative regulation of Cd^2+^ tolerance	[15,25,50,51,52,53]
*AtCNGC12*	Pathogen defence; programmed cell death	[15,50,51,52,53]
*AtCNGC13*	Pb^2+^ uptake; negative regulation of Pb^2+^ tolerance	[25]
*AtCNGC14*	Root hair growth; gravitropism; auxin signalling	[43,44,54,55,56,57]
*AtCNGC15*	Pb^2+^ and Cd^2+^ uptake; Pb^2+^ tolerance; root development	[25,58]
*AtCNGC16*	Heat and drought tolerance in pollen; negative regulation of Cd^2+^ tolerance	[25,59]
*AtCNGC17*	Growth regulation; salt tolerance	[42,60]
*AtCNGC18*	Pollen tube growth and guidance	[6,46,61,62,63]
*AtCNGC19*	Response to salt; Pb^2+^ and Cd^2+^ uptake; negative regulation of Pb^2+^ tolerance; herbivory response; pathogen defence; endophyte response; regulating cell death	[25,64,65,66,67]
*AtCNGC20*	Response to salt; pathogen defence; regulating cell death	[65,67]

**Table 2:** Examples of transport characteristics and response to cNMPs of CNGCs when either heterologously expressed or present in native membrane. Most studies have focused on the CNGCs of *Arabidopsis thaliana* (AtCNGC). However, there are also studies reporting the activity of CNGCs in *Physcomitrella patens* (PpCNGC), *Hordeum vulgare* (HvCNGC), *Oryza sativa* (OsCNGC) and *Medicago truncatula* (MtCNGC) as detailed below. “Whole cell” refers to the recording configuration in which channel activity is captured from effectively the entire plasma membrane. “Inside-out patch” refers to the configuration in which the cytosolic face of a membrane patch (held in the electrode tip) faces the bathing medium. “Cell attached” refers to the configuration in which the membrane patch (held in the electrode tip) remains undetached from the remaining membrane.

CNGCs	System	Tested Cations	Tested with cNMPs?	Results	References
AtCNGC1	HEK293— whole cell	Tested K^+^ and Na^+^ conductance	Yes	Application of 100 μM db-cAMP stimulated AtCNGC1 K^+^ and Na^+^ conductance. No K^+^ or Na^+^ conductance was observed in the absence of db-cAMP.	**[85]**
AtCNGC1	Yeast	Tested Ca^2+^ uptake	No	In the presence of yeast pheromone α factor, AtCNGC1 in a Ca^2+^ uptake-deficient yeast mutant increased colony growth, indirectly demonstrating Ca^2+^ conduction.	**[24]**
AtCNGC1, AtCNGC2, AtCNGC4	Yeast	Tested K^+^ uptake	Yes	Addition of 100 μM db-cAMP stimulated growth of a K^+^ uptake-deficient yeast mutant expressing *AtCNGC1*, *AtCNGC2*, and *AtCNGC4*.	**[86]**
AtCNGC1, AtCNGC2, AtCNGC4	Yeast	Tested K^+^ and Ca^2+^ uptake	Yes	*AtCNGC1M2* (deletion in C-terminal domain) expression in a Ca^2+^ uptake yeast mutant resulted in growth, indicating Ca^2+^ permeability of AtCNGC1. Expression of *AtCNGC2* and *AtCNGC4* enhanced growth of a K^+^ uptake-deficient yeast mutant. Application of 100 μM db-cAMP increased growth of yeast mutant transformed with *AtCNGC1M2*.	**[87]**
AtCNGC2	Yeast	Tested K^+^ uptake	Yes	In the presence of 10 μM db-cAMP or db-cGMP, transfection with *AtCNGC2* enhanced growth of a K^+^ uptake-deficient yeast mutant.	**[1]**
AtCNGC2	*Xenopus* oocytes— whole cell	Tested K^+^ conductance	Yes	Application of 10 μM db-cAMP stimulated AtCNGC2 K^+^ conductance. No K^+^ conductance was observed in the absence of db-cNMPs.	**[1]**
AtCNGC2	*Xenopus* oocytes— whole cell	Tested K^+^, Na^+^, Li^+^, Cs^+^ and Rb^+^ conductance	Yes	In the presence of 100 μM db-cAMP, AtCNGC2 conducted K^+^, Li^+^, Cs^+^ and Rb^+^. Na^+^ conductance was significantly less. No data were reported concerning conductance in the absence of db-cAMP.	**[2]**
AtCNGC2	*Xenopus* oocytes— inside-out patch	Tested K^+^ conductance	Yes	Application of 100 μM cAMP stimulated AtCNGC2 K^+^ conductance. No K^+^ conductance was observed in the absence of db-cAMP.	**[2]**
AtCNGC2	*Xenopus* oocytes— inside-out patch	Tested K^+^ and Na^+^ conductance	Yes	Application of 100 μM cAMP stimulated AtCNGC2 K^+^ conductance, but not Na^+^ conductance. No K^+^ or Na^+^ conductance was observed in the absence of cAMP. Mutation of N416 and D417 in the pore resulted in Na^+^ conductance similar to K^+^ conductance.	**[85]**
AtCNGC2	HEK293— whole cell and inside-out patch	Tested K^+^ and Na^+^ conductance	Yes	Application of 100 μM db-cAMP stimulated AtCNGC2 K^+^ conductance, but not Na^+^ conductance. No K^+^ or Na^+^ conductance was observed in the absence of db-cAMP. Mutation of N416 and D417 in the pore region resulted in Na^+^ conductance similar to K^+^ conductance.	**[2,85]**
AtCNGC2	Guard cell protoplasts— whole cell	Tested Ba^2+^ conductance (as a proxy for Ca^2+^)	Yes	Application of 1 mM db-cAMP stimulated AtCNGC2-dependent Ca^2+^ conductance.	**[30]**
AtCNGC2	HEK293T— whole cell	Tested Ca^2+^ conductance	Yes	Application of 200 μM 8Br-cAMP stimulated AtCNGC2 Ca^2+^ conductance. No data were reported concerning Ca^2+^ conductance in the absence of db-cAMP.	**[38]**
AtCNGC2, AtCNGC4	Mesophyll cell protoplasts— whole cell	Tested Ba^2+^ conductance (as a proxy for Ca^2+^)	No	Wild-type mesophyll cell protoplasts conducted Ca^2+^ in response to H_2_O_2_ or flg22. Ca^2+^ conductance was lost in *Atcngc2* or *Atcngc4* loss-of function mutants, as well as the *Atcngc2 Atcngc4* double mutant.	**[29]**
AtCNGC2, AtCNGC4	*Xenopus* oocytes— whole cell	Tested Ca^2+^, Mg^2+^, Ba^2+^, Sr^2+^, K^+^ and Na^+^ conductance	No	Independently, AtCNGC2 or AtCNGC4 did not conduct Ca^2+^ in the absence of cNMPs. Co-expression of *AtCNGC2* and *AtCNGC4* produced Ca^2+^, Sr^2+^, Ba^2+^ and K^+^-permeable (Na^+^ and Mg^2+^-impermeable) channels in the absence of cNMPs.	**[29]**
AtCNGC3	Yeast	Tested Na^+^ and K^+^ uptake	No	Yeast expressing *CNGC3* accumulated more Na^+^ and K^+^, suggesting a pathway for Na^+^ and K^+^ transport.	**[40]**
AtCNCG4	*Xenopus* oocytes— inside-out patch	Tested K^+^, Na^+^ and Cs^+^ conductance	Yes	Application of 500 μM cAMP or cGMP stimulated AtCNGC4 K^+^, Na^+^ and Cs^+^ conductance. Compared to K^+^, outward conductance of Cs^+^ was significantly lower. No conduction of K^+^, Na^+^ or Cs^+^ was observed in the absence of cNMPs.	**[41]**
AtCNGC5, AtCNGC6	Guard cell protoplasts— whole cell	Tested Mg^2+^, Ba^2+^ and Ca^2+^ conductance	Yes	Application of 500 μM 8Br-cGMP stimulated Mg^2+^, Ca^2+^ and Ba^2+^ conductance. Mg^2+^ conductance was lost in *Atcngc5 Atcngc6* double mutants. AtCNGC1, AtCNGC2 and AtCNGC20 did not appear to contribute to these guard cell 8Br-cGMP-activated currents.	**[4]**
AtCNGC5, AtCNGC6	HEK293T— whole cell	Tested Ca^2+^ and Na^+^ conductance	No	HEK293 cells expressing *CNGC5* or *CNGC6* displayed inward currents carried by Ca^2+^, not Na^+^.	**[43]**
AtCNGC6	Root protoplasts— whole cell	Tested Ca^2+^ conductance	Yes	Application of 50 μM db-cAMP stimulated AtCNGC6-dependent Ca^2+^ conductance, application of a phosphodiesterase inhibitor also stimulated Ca^2+^ conductance.	**[45]**
AtCNGC7, AtCNGC8	*Xenopus* oocytes— whole cell	Tested Ca^2+^ conductance	No	AtCNGC7 or AtCNGC8 Ca^2+^ conductivity was not observed in the absence of cNMPs.	**[46]**
AtCNGC7, AtCNGC8, AtCNGC9, AtCNGC10, AtCNGC16 and AtCNGC18	HEK293T— whole cell	Tested Ca^2+^ and K^+^ conductance	Yes	Application of 100 μM 8Br-cAMP or 100 μM 8Br-cGMP stimulated AtCNGC7, AtCNGC8, AtCNGC9, AtCNGC10, AtCNGC16 and AtCNGC18 Ca^2+^ conductance. Ca^2+^ conductance did not require 8Br-cNMP application. No significant K^+^ conductance reported.	**[6]**
AtCNGC10	*E. coli* and yeast	Tested K^+^ uptake	Yes	AtCNGC10 complemented *E. coli* and yeast K^+^ uptake mutants. In *E. coli*, co-expression of *AtCNGC10* and *CaM* inhibited cell growth, but cGMP overcame this.	**[88]**
AtCNGC10	HEK293— whole cell	Tested K^+^ conductance	Yes	In the presence of 100 μM db-cGMP, AtCNGC10 conducted K^+^. No data were reported concerning conductance in the absence of db-cAMP.	**[80]**
AtCNGC10	Yeast	Tested K^+^ and Na^+^ uptake	No	*AtCNGC10*-transformed yeast accumulated more Na^+^ in the presence of 20 mM NaCl. Expression rescued growth of K^+^ uptake-deficient yeast.	**[49]**
AtCNGC11, AtCNGC12	Yeast	Tested K^+^ uptake	Yes	Growth of K^+^ uptake-deficient yeast was complemented by AtCNGC11, AtCNGC12 or the chimeric AtCNGC11/12. Growth was enhanced by 100 μM db-cAMP but not db-cGMP.	**[53]**
AtCNGC11, AtCNGC12	Yeast	Tested Ca^2+^ uptake	No	Expression of *AtCNGC11*, *AtCNGC12* or *AtCNGC11/12* complemented growth of Ca^2+^ uptake-deficient yeast.	**[51]**
AtCNGC11, AtCNGC12	Yeast	Tested K^+^ uptake	No	AtCNGC11/12 or AtCNGC12 restored growth of K^+^ uptake-deficient yeast.	**[81]**
AtCNGC11, AtCNGC12	*Xenopus* oocytes— whole cell	Tested Ca^2+^ conductance	Yes	Expression of *AtCNGC12* caused a Ca^2+^ conductance that was not enhanced by cNMPs. *AtCNGC11* expression did not cause a Ca^2+^ conductance, even with cNMPs. Co-expression did not affect the AtCNGC12-dependent conductance.	**[8]**
AtCNGC14	*Xenopus* oocytes— whole cell	Tested Ca^2+^ conductance	No	AtCNGC14 Ca^2+^ conductivity was observed in the absence of cNMPs. It was not tested whether application of cNMPs would increase Ca^2+^ conductance.	**[57]**
AtCNGC14	*Xenopus* oocytes— whole cell	Tested Ca^2+^ conductance	No	AtCNGC14 inward Ca^2+^ currents were observed in the absence of cNMPs.	**[56]**
AtCNGC18	*E. coli*	Tested Ca^2+^ uptake	No	Expression of *AtCNGC18* in *E. coli* caused Ca^2+^ accumulation.	**[62]**
AtCNGC18	HEK293T— whole cell	Tested Ca^2+^ conductance	Yes	Application of 100 μM 8Br-cAMP or 100 μM 8Br-cGMP stimulated greater AtCNGC18 Ca^2+^ conductance, but not 20 μM 8Br-cNMP. Ca^2+^ conductance did not require 8Br-cNMP application.	**[5]**
AtCNGC18	*Xenopus* oocytes— whole cell	Tested Ca^2+^ conductance	Yes	In the presence of 100 μM db-cAMP, AtCNGC18 conducted Ca^2+^. No data were reported concerning conductance in the absence of db-cAMP.	**[63]**
AtCNGC18	Pollen tube protoplasts— whole cell	Tested Ca^2+^ conductance	Yes	Application of 100 μM 8Br-cGMP stimulated AtCNGC18-dependent Ca^2+^ conductance. Ca^2+^ conductance was not apparent in the absence of 8Br-cGMP.	**[6]**
AtCNGC18	*Xenopus* oocytes— whole cell	Tested Ca^2+^ conductance	No	AtCNGC18 Ca^2+^ conductivity was observed in the absence of cNMPs. Co-expression of AtCNGC18 with AtCNGC7 or AtCNGC8 eliminated Ca^2+^ conductivity.	**[46]**
AtCNGC19	*Xenopus* oocytes— whole cell	Tested Ca^2+^, Na^+^ and K^+^ conductance	Yes	In the presence of 300 μM db-cAMP, AtCNGC19 elicited Ca^2+^ inward currents but not K^+^ and Na^+^ currents.	**[66]**
AtCNGC19, AtCNGC20	*Xenopus* oocytes— whole cell	Tested Ca^2+^ conductance	No	AtCNGC19 and AtCNGC20 conductivity was observed in the absence of cNMPs. It was not tested whether application of cNMPs would increase Ca^2+^ conductance. Co-expression of *AtCNGC19* and *AtCNGC20* increased conductance compared to independently expressed *AtCNGC19* and *AtCNGC20*.	**[67]**
PpCNGCb	Moss protoplasts cell attached	Tested Ba^2+^ conductance (as a proxy for Ca^2+^)	No	Ba^2+^ conductivity did not require application of cNMPs, but it is possible that there were endogenous cNMPs. Ba^2+^ conductivity was altered in *Ppcngcb* mutants.	**[28]**
HvCNGC2-3	*Xenopus* oocytes— whole cell	Tested K^+^ and Na^+^ conductance	Yes	Application of 10 μM 8Br-cGMP stimulated HvCNGC2-3 Na^+^ and K^+^ conductivity only in the co-presence of both ions. No Na^+^ or K^+^ conductance was observed in the absence of cGMP, or 10 μM 8Br-cAMP. Ca^2+^ conductivity was not observed.	**[7]**
OsCNGC9	HEK293— whole cell	Tested Ca^2+^ and K^+^ conductance	No	OsCNGC9 Ca^2+^ conductivity was observed in the absence of cNMPs. Comparatively little K^+^ conductivity was observed. It was not tested whether application of cNMPs would increase Ca^2+^ or K^+^ conductance.	**[89]**
OsCNGC13	HEK293— whole cell	Tested Ca^2+^ and K^+^ conductance	No	OsCNGC13 mediated Ca^2+^ inward currents but not K^+^ currents. It was not tested whether application of cNMPs would increase Ca^2+^ conductance.	**[90]**
MtCNGC15	*Xenopus* oocytes— whole cell	Tested Ba^2+^ and Ca^2+^ conductance	No	MtCNGC15 Ca^2+^ conductivity did not require application of cNMPs. It was not tested whether application of cNMPs would increase Ca^2+^ conductance.	**[77]**

## Data Availability

Data can be obtained by contacting the corresponding author.

## Abbreviations

AC	Adenylyl cyclase
AGN	Alanine-glycine-asparagine
AHA	Arabidopsis H^+^-ATPase
AND	Alanine-asparagine-aspartic acid
Apo-CaM	Calmodulin without Ca^2+^ ligands
At	Arabidopsis thaliana
BAK1	BRI-associated receptor kinase1
BiFC	Bifluorescence complementation
Bo	Brassica oleracea
Bra	Brassica rapa
BRI1	Brassinosteroid insensitive1
Ca^2+^/CaM	Calmodulin with Ca^2+^ ligands
CaM	Calmodulin
CaMBD	Calmodulin-binding domain
cAMP	Cyclic adenosine monophosphate
cGMP	Cyclic guanosine monophosphate
CNDB	Cyclic nucleotide-binding domain
CNGC	Cyclic nucleotide-gated channel
CNGCL	A gene or protein which contains some, but not all the domains associated with cyclic nucleotide-gated channels
cNMP	Cyclic nucleotide monophosphate
CPK32	Calcium-dependent protein kinase 32
CT	Carboxy-terminal domain
db-cAMP	Dibutyryl-cyclic adenosine monophosphate
db-cGMP	Dibutyryl-cyclic guanosine monophosphate
DMI1	Does not make infections 1
flg22	Flagellin 22 peptide
FLS2	Flagellin Sensitive 2
GC	Guanylyl cyclase
GNL	Glycine-asparagine-leucine
GQG	Glycine-glutamine-glycine
GQN	Glycine-glutamine-asparagine
GQS	Glycine-glutamine-serine
GUS	β-glucuronidase
HEK293	Human embryonic kidney cell line 293
Hv	Hordeum vulgare
IQ	Isoleucine-glutamine
KUP	K^+^ uptake permease
Lj	Lotus japonicus
LRRAC1	Leucine-rich repeat adenylyl cyclase1
Mt	Medicago truncatula
Ntab	Nicotiana tabacum
NOGC1	Nitric oxide-dependent guanylate cyclase1
NT	N-terminal domain
Os	Oryza sativa
OSCA1.3	hyperOsmolality-induced [Ca^2+^]i increase 1.3
PepR1	Plant elicitor peptide recptor1
Pp	Physcomitrella patens
PSKR1	Phytosulfokine receptor1
RLCK185	Receptor-like cytoplasmic kinase 185
SERK4	Somatic embryogenesis receptor kinase 4
Sl	Solanum lycopersicum
WAKL10	Wall-associated kinase (WAK)-like 1
Zj	Ziziphus jujube
